# A Cell Cycle Role for the Epigenetic Factor CTCF-L/BORIS

**DOI:** 10.1371/journal.pone.0039371

**Published:** 2012-06-19

**Authors:** Manuel Rosa-Garrido, Laura Ceballos, Pilar Alonso-Lecue, Cristina Abraira, M. Dolores Delgado, Alberto Gandarillas

**Affiliations:** 1 Cell Cycle, Stem Cell Fate and Cancer Laboratory, Fundación Marqués de Valdecilla-Instituto de Formación e Investigación Marqués de Valdecilla, Santander, Spain; 2 Departamento de Biología Molecular, Instituto de Biomedicina y Biotecnología de Cantabria, Universidad de Cantabria-Consejo Superior de Investigaciones Científicas, SODERCAN, Santander, Spain; 3 Institut National de la Santé et de la Recherche Médicale, ADR Languedoc-Roussillon, Montpellier, France; Florida State University, United States of America

## Abstract

CTCF is a ubiquitous epigenetic regulator that has been proposed as a master keeper of chromatin organisation. CTCF-like, or BORIS, is thought to antagonise CTCF and has been found in normal testis, ovary and a large variety of tumour cells. The cellular function of BORIS remains intriguing although it might be involved in developmental reprogramming of gene expression patterns. We here unravel the expression of CTCF and BORIS proteins throughout human epidermis. While CTCF is widely distributed within the nucleus, BORIS is confined to the nucleolus and other euchromatin domains. Nascent RNA experiments in primary keratinocytes revealed that endogenous BORIS is present in active transcription sites. Interestingly, BORIS also localises to interphase centrosomes suggesting a role in the cell cycle. Blocking the cell cycle at S phase or mitosis, or causing DNA damage, produced a striking accumulation of BORIS. Consistently, ectopic expression of wild type or GFP- BORIS provoked a higher rate of S phase cells as well as genomic instability by mitosis failure. Furthermore, down-regulation of endogenous BORIS by specific shRNAs inhibited both RNA transcription and cell cycle progression. The results altogether suggest a role for BORIS in coordinating S phase events with mitosis.

## Introduction

CTCF is a Zinc finger DNA binding protein initially identified as a transcriptional regulator [1] and later established as a chromatin insulator binding protein [2]. CTCF has attracted much attention in the last years since it has been associated with heritable genomic imprinting [2], [3] and it has been proposed as a master keeper of global chromatin structure [4], [5]. The essential role for CTCF in genome regulation was revealed by genome-wide analysis [6], [7], [8], [9].

CTCF-like protein BORIS (Brother of the Regulator of Imprinted Sites; or CTCFL) has been proposed to be a CTCF antagonist [10]. The BORIS protein (663 aminoacids) exhibits high homology with CTCF in the central domain containing 11 Zinc-Finger elements, where every amino acid relevant to DNA binding is exactly the same. CTCF and BORIS might thus bind to the same DNA target sequences. On the contrary, the flanking N- and C- terminal regions show very little sequence homology between of BORIS and CTCF, implying that they may recruit different associated cofactors [11], [12], [13].

BORIS was originally found only in spermatocytes within normal tissues [11]. More recently, it has been detected in human oocytes, ovary, embryonic stem cells [14] and various foetal tissues [15]. Consistent with its significant level of expression in testis, BORIS knock-out mice suffer from spermatogenesis defects that result in small testes [16]. In addition, BORIS is aberrantly expressed in many tumours [17], [18], [19] and was thus defined within the cancer-testis group of genes [10]. Because of the high homology of the zinc fingers domain, BORIS is thought to bind to the same DNA sequences as CTCF [10]. However, CTCF and BORIS differ significantly in their amino and carboxy termini, suggesting that they may act differently by recruitment of different associated cofactors [11], [13]. They are thought to be antagonists also because of the mutually exclusive manner of their distribution during male germ cell development, although they are aberrantly co-expressed in cancer cells. CTCF has been considered as a tumour suppressor (reviewed in [20], it inhibits cell growth when ectopically expressed [10], [21], [22], it is ubiquitously distributed in somatic cells and it is altered in a number of tumours through genetic and epigenetic mechanisms [23], [24]. It is unclear whether aberrant expression of BORIS interferes in tumour cells with the normal function of CTCF [10], or it elicits CTCF independent functions.

The regulation of BORIS is a complex promoter- and cell type-dependent process [25]. 23 differentially expressed isoforms of BORIS have recently been reported [15]. Although the biological data available have suggested a role for BORIS in epigenetic genome reprogramming in testis [11] and in the proliferation of cancer cells [17], little is known about the mechanisms eliciting these functions. In part this issue has been hampered by the restricted detection of the BORIS protein in normal tissues. In order to gain insight into this issue we have studied a primary human system. After running a small scale screening on a panel of cell lines and tissues, we detected BORIS mRNA in skin samples.

The epidermis is a stratified epithelium that self-renews throughout adult life from the stem cells in the basal layer [26]. As keratinocytes differentiate terminally, they cease proliferation and migrate through the suprabasal layers. During this process keratinocytes evolve from quiescent stem cells to actively proliferating cells and subsequently, to actively metabolic differentiating cells. Keratinocyte differentiation involves a significant cell mass increase and high production of RNA and proteins [27], [28] and requires continuous reprogramming of gene expression and chromatin remodelling [29].

We have explored the distribution of CTCF and BORIS in human epidermis in situ and in primary keratinocytes in vitro. We show a striking localisation of BORIS to the nucleolus and other areas of active transcription and to interphase centrosomes. This localisation was confirmed in mouse testis and a panel of cell lines from diverse origins. By ectopic expression or inactivation studies, we also demonstrate a function of BORIS in RNA transcription, cell cycle progression and genomic instability. We discuss the potential implications of this novel function of BORIS for cancer cells.

## Materials and Methods

### Skin Biopsies and Cell Culture, Treatments and Transfections

Ethics Statement: Ethical permission for this study was demanded, approved and obtained from the Ethical Committee for Clinical Research of the Cantabria Council, Spain (FIS-08/0890). In all cases, human tissue material to discard after surgery was obtained with informed written consent presented by clinicians to the patients or the parents/guardians of minors involved in the study, the identity was not kept and the material was thus treated completely anonymously.

Skin biopsies were provided by the Plastic Surgery and Paediatrics Services of clinic St Jean (Montpellier, France) and of Hospital Marqués de Valdecilla (Santander, Spain) from circumcisions (neonatal foreskin) or from plastic surgery (adult breast, scalp). The fat and most of the connective tissue was removed using curved scissors, and the epidermis was embedded in OCT compound (Tissue-Tek, Sakura) and submerged in liquid Nitrogen (Thermo Shandon). Frozen tissue was microsectioned and 5–10 µm thick sections were collected on Superfrost Plus glass-slides (Thermo Scientific) and air dried [30].

Primary keratinocytes were isolated from neonatal human foreskin and cultured in the presence of a mouse fibroblast feeder layer (inactivated by a 2 h treatment with 4 µg/ml mitomycin C), 10% serum and 1.2 mM Ca+2, as described [31], [32].

Primary keratinocytes were treated for 48 hours with cdk inhibitors or mitosis kinase inhibitors: 50 mM of hydroxyurea (Tocris Bioscience), 1 mM of the Aurora B Kinase inhibitor ZM447439 (Tocris Bioscience) or 100 nM of the Polo Like Kinase inhibitor BI2536 (Axon MedChem BV). Parallel control cultures were always subjected to the DMSO vehicle.

HEK293T (human embryonic kidney; [33]), HeLa (human cervical cancer; [34]), HCT116 (human colorectal cancer; [35]), MCF7 (human breast cancer; [36]), MEF-1 (obtained from the American Type Culture Collection) and 3T3-J2 (mouse embryonic fibroblasts; [31]) cell lines were grown in DMEM supplemented with 10% foetal calf serum (FCS; LONZA), 150 µg/ml of gentamycin and 2 µg/ml of ciprofloxacin at 37°C in a humidified 5% CO_2_ atmosphere. HEK293T cells were transfected by the use of the jetPEI transfection reagent (poly Plus) as indicated by the manufacturer, with the following constructs: pEGFP-CTCF [37], pCDNA-CTCF [22], pEGFP-BORIS, pCMV-BORIS (kindly provided by Elena Klenova, University of Essex, UK), pEGFP-BORIS-ZF-domain (kindly provided by Niels Galjart and Frank Sleutels, Erasmus MC, Rotterdam, Netherlands), Fibrillarin-Cherry (kindly gift by Prof. Angus I. Lamond, University of Dundee, Dundee, UK; [38]) or shRNAs BORIS (Genecopoieia Inc, MD, USA; see below).

### Mice Tissue

Mice in a C57BL/6 background were obtained from Harland Ibérica (Barcelona, Spain) and used to purify RNA from different tissues for quantitative RT-PCR experiments. All experiments were performed with 10–12 week-old animals and approved by the Universidad de Cantabria Institutional Laboratory Animal Care and Use Committee (Ref. Numb. 2008/07).

### RNA Purification and RT-PCR Analysis

Total RNA was extracted from human cell lines and different mouse and human tissues using Trizol reagent (Invitrogen). 2 µg of RNA were used for the reverse transcription with the iScript cDNA Synthesis Kit (Bio-Rad). cDNA was amplified using specific primers (see Information S1) and Qiagen Quantitect Sybr Green mix with an iQ5 Real-Time PCR Detection System (Bio-Rad). Amplification efficiencies, determined by amplifying log dilutions of plasmids containing the corresponding coding sequences, were near 100%. For semiquantitative PCR, a Thermal Cycler C1000 (Bio-Rad) was used. The PCR conditions were determined depending on the nature and complexity of the primers. The results were normalised to β-actin for human samples and to GAPDH for mouse samples using the comparative DeltaDeltaCt (ΔΔCt) method.

### Chromatin Immunoprecipitation (ChIP) Assays

ChIP assays were performed using a modified version of the Upstate Biotechnology protocol. Briefly, 5×10^6^ cells were fixed in 1% formaldehyde, lysed in lysis buffer (50 mM Tris-HCl pH 8, 10 mM EDTA, 1% SDS, protease inhibitor cocktail Set I) and sonicated using a Bioruptor UCD-200 (Diagenode), leading to fragments between 250 and 1000 bp. ChIP was performed using Dynabeads-protein G (Invitrogen) coupled to anti-BORIS antibody (abcam ab18337) or anti-GFP antibody (Invitrogen A11122). The DNA recovered was purified (Qiaquick columns, Qiagen) and analysed by quantitative real time-PCR as described above. PCR was performed in duplicate with equal amounts of specific antibody immunoprecipitated sample, control (IgG) and Input. Primers used for H 42.1 and H 37.9 sites in human ribosomal DNA and amplification conditions were previously described [39]. Values were normalised to input measurements and enrichment was calculated using the comparative Ct method.

### Immunofluorescence and Confocal Microscopy

For immunofluorescence assays, keratinocytes and adherent cell lines were grown on glass coverslips. Cells and skin sections were fixed and permeabilized with cold methanol −20°C) for 10 min, washed with PBS and successively incubated with primary antibodies and secondary antibodies. Primary antibodies used were: anti-CTCF (Abcam ab10571, ab84372 or Upstate 07-729); anti-BORIS (Rockland 600-401-907; Abcam ab18337 or a chicken polyclonal antibody [17]; anti-keratin K10 (Sigma WH0003858M1); anti-keratins K1,10,11 (American Research Products, Inc 03-61808), anti-involucrin (Sigma I-9018); anti-Pan Histone (Roche 1492-519); anti-γTubulin (Sigma T-6557); anti-UBF (Santa Cruz sc-13125); anti-Fibrillarin (Abcam ab4566). Secondary antibodies (Jackson InmunoResearch Laboratories) were conjugated with Texas Red, fluorescein isothiocyanate or Cy5. Parallel control samples with no primary antibody, a negative primary antibody anti-CD8 (Sigma C-7423); mouse IgGs (Santa Cruz sc-2027) or rabbit antiserum, showed unspecific fluorescent staining.

Nucleic acids were visualised with the red dye propidium iodide (4 µg/ml final concentration) on fixed and permeabilized cells. For detection of GFP-CTCF and GFP-BORIS fusion proteins, transfected HEK293T cells were fixed with cold methanol (–20°C), washed and mounted with anti-fading mounting medium Vectashield (Vector Laboratories) with 4′,6′-Diamidino-2-phenylindole dihydrochloride (DAPI) to visualize the nucleus. Cell samples were examined and images acquired using a Zeiss IMAGER M1 fluorescence microscope and a Zeiss LSM 510 META confocal laser microscope equipped with argon (488 nm) and HeNe (543 nm) and HeNe (633 nm) lasers. Z-stack digital images were reconstructed after collecting 40 frames by a confocal microscope (Nikon A1R) at every 0,223 µm depth and processing by Nis elements AR, 3.2 64 bits).

Cell counting for cell cycle markers after transfection with GFP-constructs was performed by scoring positive cells of the given marker within the GFP positive or negative populations, taken >1000 total cells and >100 GFP positive cells per sample from 4 different fields (micro-photographs) in duplicates.

### Immunoblotting

Primary keratinocytes were lysed with a 7 M urea buffer (0.1 M Tris-HCl pH 6.8; 7 M urea; 4% SDS; 10% 2-mercaptoethanol, 0.1% bromo phenol blue) and protein levels were determined by immunoblot as described [22]. The membranes were incubated with primary antibodies anti-CTCF (BD Biosciences 612148) or anti-BORIS (Rockland 600-401-907 or abcam ab18337) and then with fluorescent secondary antibodies (IRDye Antibodies, Li-COR, Biosciences). The immunocomplexes were detected with an Odyssey Infrared Imaging System (Li-COR, Biosciences). For protein loading control, the blots were re-stained with anti-β-actin antibody (I-19 Santa Cruz Biotech. sc-1616).

### Run-on Transcription Assay and BrdU Incorporation

For immunodetection of nascent RNA, short pulses of 5′-fluorouridine (5′-FU; Sigma) were performed on primary keratinocytes, generally as described in [40]. Primary keratinocytes were cultured directly on glass coverslips and 5′-FU was added to a final concentration of 2 mM in the culture medium. After 5 or 10 min, cells were fixed with 3.7% paraformaldehyde in HPEM buffer (30 mM Hepes, 65 mM Pipes, 2 mM EGTA, 2 mM MgCl2) containing 0.5% Triton X-100 for 10 min The incorporation of 5′-FU into nascent RNA was detected with an antibody against halogenated UTP (anti-BrdU clone BU-33 Sigma) and a Texas Red-conjugated secondary antibody (Jackson Laboratories).

To monitor DNA synthesis in live cells, bromodeoxyuridine (BrdU) incorporation assays were performed. Primary keratinocytes growing on glass coverslips were pulsed with 40 µg/ml BrdU (Roche) for 15 min, washed in HPEM buffer and fixed with 3.7% paraformaldehyde as above. Cells were treated with HCl, neutralized and sequentially incubated with anti-BrdU antibody and Texas Red-conjugated secondary antibody. Cells were washed and mounted with anti-fading mounting medium with DAPI (Vector Laboratories).

### Knock-down of Endogenous BORIS with shRNAs

shRNA constructs targeting BORIS were from Genecopoieia Inc (Rockville, MD, USA; HSH003033-HIVH1). Four different plasmids with different target sequences for BORIS (sh1, OS245161; sh2 OS245162, sh3 OS245163 and sh 4 OS245164) were transiently transfected into HEK293T cells using JetPEI (Polyplus transfection). Cells were analysed 48 hours after transfection. Quantification of mRNA transcripts was performed using RT-PCR and all data normalized to ribosomal protein S14. The most efficient constructs in diminishing BORIS expression were selected (sh2 and sh4). A construct carrying a scrambled sequence of a similar size was used as a control. All shRNAs were associated to a GFP control to visualise transfected cells.

### Flow-cytometry Analyses

Trypsinized HEK 293T cells were fixed in cold 70% ethanol and stained with propidium iodide as described [41]. After staining, cells were firmly resuspended and filtered through a 70 µM mesh to minimize the presence of aggregates and then analysed on a BD FACSCanto™. 10 000 events were gated and acquired in list mode for every sample.

### Clonogenic Growth Assay

After transfection, HEK 293T cells were placed in 6-well tissue culture plates at a density of 10^4^ cells/well. 14 days later, cells were fixed and stained with Cristal violet 1% ETOH to visualize the colonies.

## Results

### CTCF and BORIS Protein Expression in Normal Human Skin

**Figure 1:**
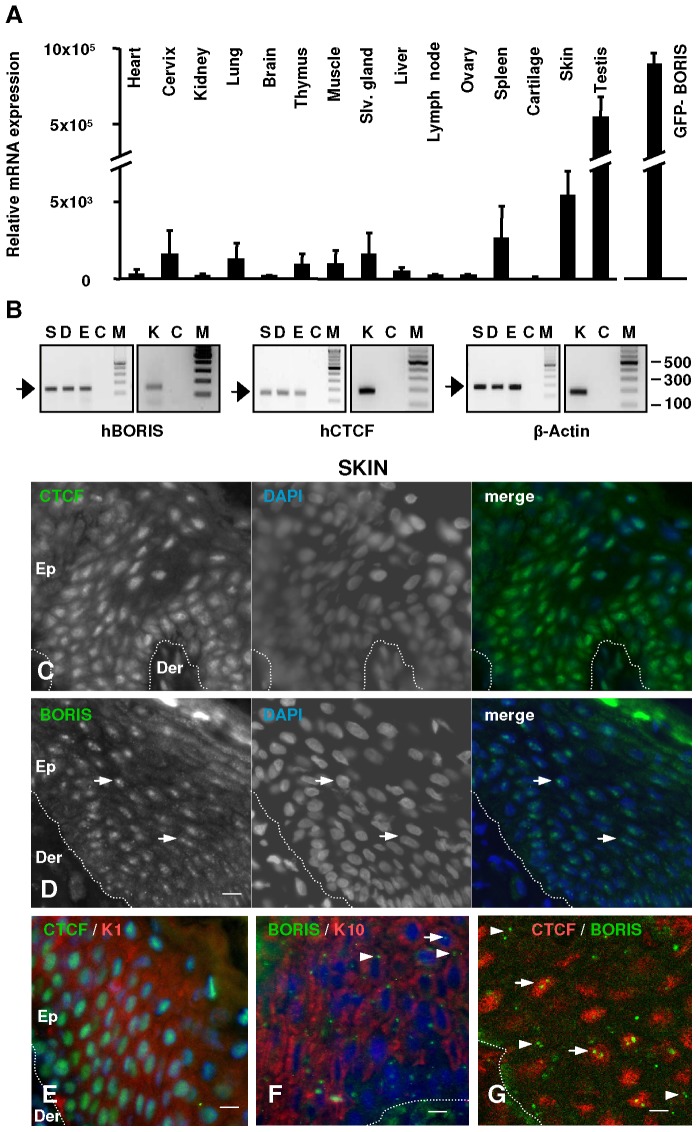
Expression of BORIS and CTCF in mouse tissues and human skin. A ) BORIS mRNA expression in mouse tissues as analysed by quantitative RT-PCR by the comparative Ct method and normalised to GAPDH. Data are represented as fold changes relative to the lowest BORIS/GAPDH ratio (cartilage, designated as 1.0). For each sample, measurements were done in duplicate using two different primer sets. Error bars represent s.d. HEK293T cells transfected with pEGFP-mBORIS were used as positive control (right graph). **B**) BORIS, CTCF and β-Actin (internal control) mRNA expression in human skin and primary keratinocytes by semiquantitative RT-PCR (H0.3 primer set was used, see Information S1). Human total skin (S), dermis (D), epidermis (E) and freshly isolated keratinocytes (K), buffer only-control (C) or molecular weight markers (M). **C,D**) Indirect immunofluorescence experiments on human skin sections with anti-CTCF or anti-BORIS antibodies as indicated. Colours as indicated. The nuclei were visualised with DAPI (blue). Dotted line indicates the basal membrane that separates the epidermis (Ep) from the dermis (Der). Scale bar: 50 µm. Photographs are representative of studies on five different human individuals with three different polyclonal antibodies for BORIS and two different antibodies for CTCF. **E,F**) Double immunofluorescence for CTCF or BORIS and markers of post-mitotic terminal differentiation keratins K1/K10. Colours as indicated. Scale bar: 20 µm. **G**) Double immunofluorescence for CTCF (red) or BORIS (green). Scale bar: 10 µm. Arrows indicate the focal accumulation of BORIS within the nuclei, arrowheads indicate BORIS dots beside the nuclei. Colours as indicated.

We analysed a panel of mouse tissues (15) for BORIS mRNA expression by quantitative real time RT-PCR. These studies identified high levels of BORIS mRNA in testis (Fig. 1A) as previously described [11]. Lower but reproducible levels of BORIS mRNA were also detected in other mouse tissues, particularly in skin and at a lesser extent in spleen (Fig. 1A). We therefore investigated BORIS mRNA expression by RT-PCR in human skin. We found significant levels in total human skin and freshly isolated whole dermis epidermis or disaggregated keratinocytes (Fig. 1B and Information S1). BORIS transcripts were detectable with the three primer sets employed, designed to amplify different regions of BORIS mRNA (Fig. 1B and Information S1). CTCF mRNA expression was also found in epidermis, dermis and isolated keratinocytes (Fig. 1B). These results suggest that BORIS is expressed in normal human skin.

In order to determine the distribution and localisation of the two proteins in the epidermis, we investigated the expression of CTCF and BORIS proteins by indirect immunofluorescence on human skin sections. We labelled skin with antibodies for BORIS or CTCF of different origins and for keratins 1 or 10 as a marker of post-mitotic terminal differentiation. Expression of CTCF protein was observed throughout the suprabasal layers of epidermis with a punctate focal pattern in the whole nucleus (Fig. 1C,E), consistent with previous reports in other cell types [37], [42], [43]. In contrast, BORIS accumulated in discrete nuclear and perinuclear spots or foci of all epidermal cells (arrows and arrowheads, Fig. 1D,F). The distribution of CTCF and BORIS in epidermis was confirmed with two and three different polyclonal antibodies, respectively (see Materials and Methods), on skin sections from more than five different individuals. Double labelling experiments for CTCF and BORIS by confocal or conventional fluorescence microscopy showed the differential distribution of the two proteins in the epidermis, whose signals did not generally co-localise within the nucleus (Fig. 1G and Information S1). It is important to note that the immunostaining intensity of CTCF diminished in the more differentiated layers of the epidermis (Fig. 1C,E and Information S1). Therefore, both proteins were detected throughout the epidermis, although they displayed a differential distribution.

### Accumulation of BORIS in the Nucleoli and other Active Transcription Sites

**Figure 2:**
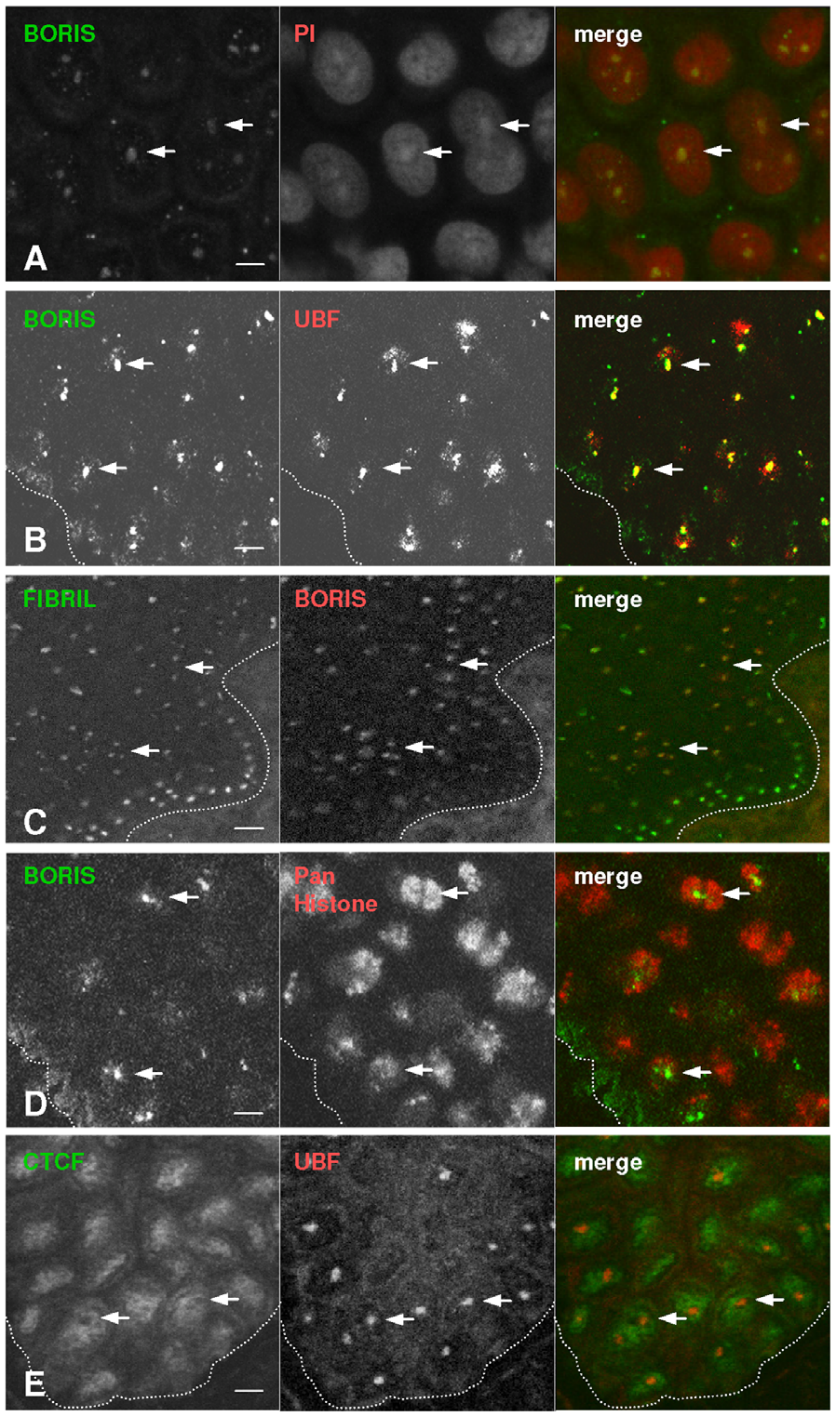
BORIS localises to the nucleoli of human epidermal cells. A–E ) Double immunofluorescence analyses were performed on human skin sections with antibodies to BORIS or CTCF and UBF, Fibrillarin or pan-histone, as indicated. Coulors as indicated. Nucleoli were counterstained with propidium iodie (PI) in A. Arrows point at nucleoli (**A–C,E**) or histone-dark areas (**D**). Note the coincidence between the BORIS protein, nucleolar markers and dark-histone areas. Scale bar: 20 µm.

The staining pattern of BORIS in epidermal keratinocytes was reminiscent of the nucleoli. This possibility was investigated by staining the epidermis with propidium iodide (PI), a cytochemical marker for nucleic acids. The nucleolus is a centre of active synthesis and processing of rRNAs [44] and references therein) and with the PI staining appears more intensely labelled than the rest of the nucleus due to its high content in rRNAs (Fig. 2A). We further performed double immunofluorescence for BORIS and nucleolar markers UBF or Fibrillarin [45]. UBF is a transcription factor of rRNA genes preferentially located in fibrillar centres of the nucleolus. It plays an important role in inducing the euchromatic state of the rDNA. Fibrillarin is involved in the processing of pre-rRNA that takes place in the nucleolus. BORIS strongly co-localised with both nucleolar markers within the epidermal nuclei (Fig. 2B,C). It is to note however that while Fibrillarin was stronger in the proliferative basal of epidermis, nucleolar expression of BORIS was stronger in the suprabasal layers of the epidermis, composed of differentiating cells that re-replicate after a mitotic block (Fig. 2C; [28], [32]). We also explored the distribution of BORIS with respect to chromatin density by double labelling with a pan-histone antibody. As shown in Fig. 2D, BORIS localised in nuclear domains that were dull for histone staining, corresponding to areas of low DNA density. A generally inverse pattern was found for CTCF, predominant in bright histone areas (not shown) and in UBF negative domains (Fig. 2E) as previously described in other cell types [37]. Taken together, the results from nucleolar markers and histone density suggest that BORIS is present in less condensed DNA regions typical of euchromatin.

**Figure 3:**
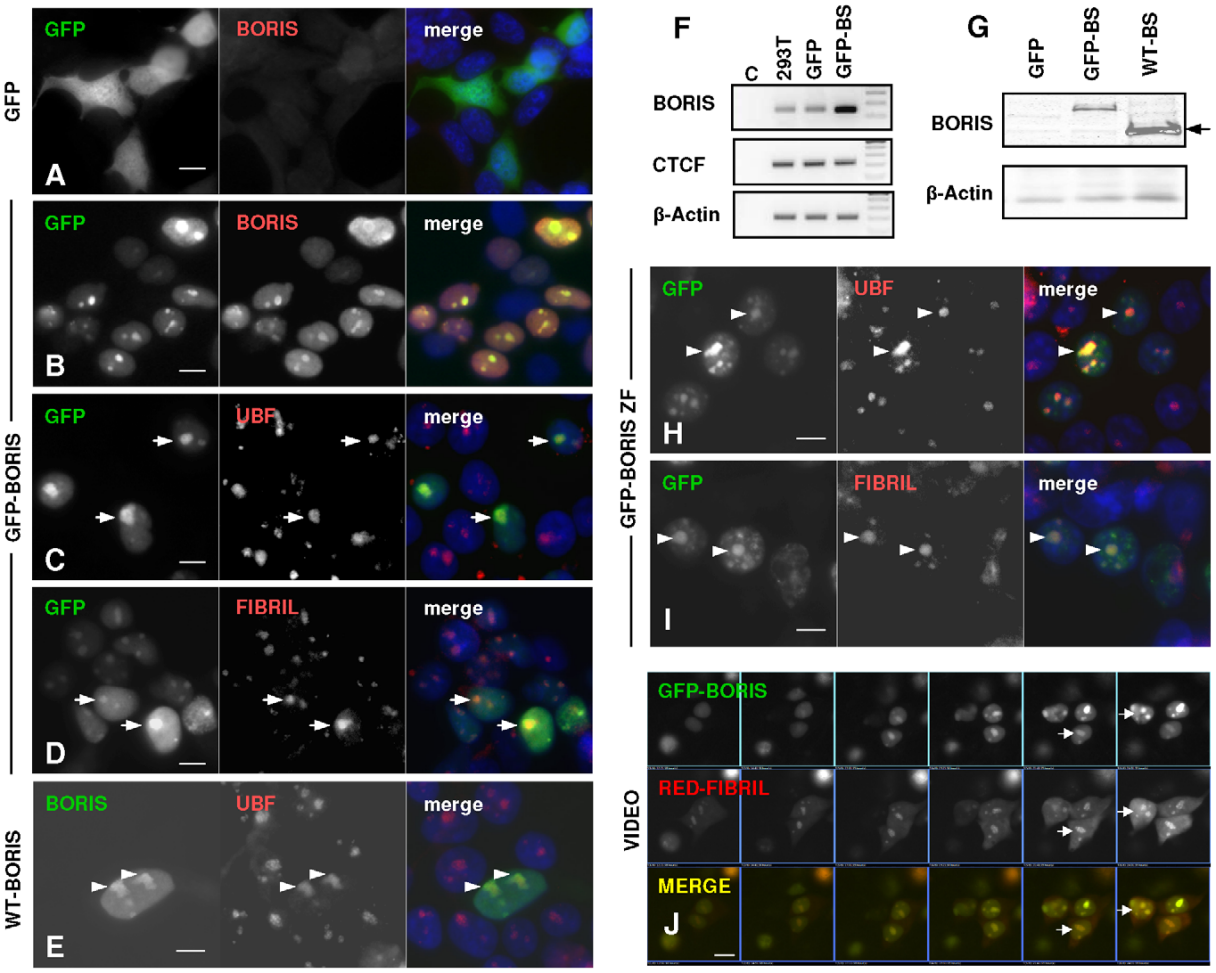
Exogenous GFP-BORIS localises to the nucleoli. A–E, H,I ) Detection of GFP (green) or BORIS (red), UBF, or Fibrillarin by immunofluorescence in HEK293T cells 24 hours after transfection with plasmids carrying GFP (A) or GFP-BORIS (B-D), wild-type BORIS (E) or GFP-Zinc finger domain of BORIS (H,I), as indicated on the left side. The nuclei were visualised with DAPI (blue). Scale bar: 10 µm. **F**) Detection of BORIS mRNA expression by RT-PCR in HEK293T cells transiently transfected as above. Primers for CTCF and β-actin were used as controls. BS: BORIS. **G**) Detection of BORIS by western blotting with the antibodies used for the immunofluorescence analyses above. Note the higher molecular weight of the fusion protein GFP-BORIS, compared to the wild-type protein (arrow). β-Actin as loading control. **J**) Video microphotograms showing the co-localisation of GFP-BORIS and Fibrillarin-Cherry in live cells after transient transfection (see also Video S1).

We aimed to further explore the nuclear localisation of BORIS, by expressing a GFP-fusion form of the protein. Since primary keratinocytes are very hard to transfect, we made use of human epithelial HEK293T cells. We performed transient transfection with GFP-BORIS and analysed its localisation after 24 h. In cells transfected with the control GFP vector the signal was distributed throughout the cell (Fig. 3A). In contrast, the GFP-BORIS fusion protein strongly localised to the nucleoli, as it was revealed by immunostaining for BORIS, UBF or Fibrillarin (Fig. 3B–D). Both the exogenous GFP-BORIS fusion protein and the wild-type protein were detected with the anti-BORIS antibodies used in the studies on skin, further confirming their specificity (Fig. 3B,E). We assessed the overexpression of BORIS protein also by RT-PCR, with various primer sets (Fig. 3F and Information S1) and by western blotting, with two different polyclonal antibodies (Fig. 3G and not shown). Interestingly, the truncated Zinc-Finger domain of BORIS was sufficient to drive nucleolar localisation (Fig. 3H,I). The GFP-CTCF protein showed a nucleoplasmic distribution. We previously reported nucleolar localisation of CTCF [37]. Finally, double transfection experiments with GFP-BORIS and Fibrillarin-Cherry (red) showed their co-localisation in live cells (Fig. 3J and Video S1).

**Figure 4:**
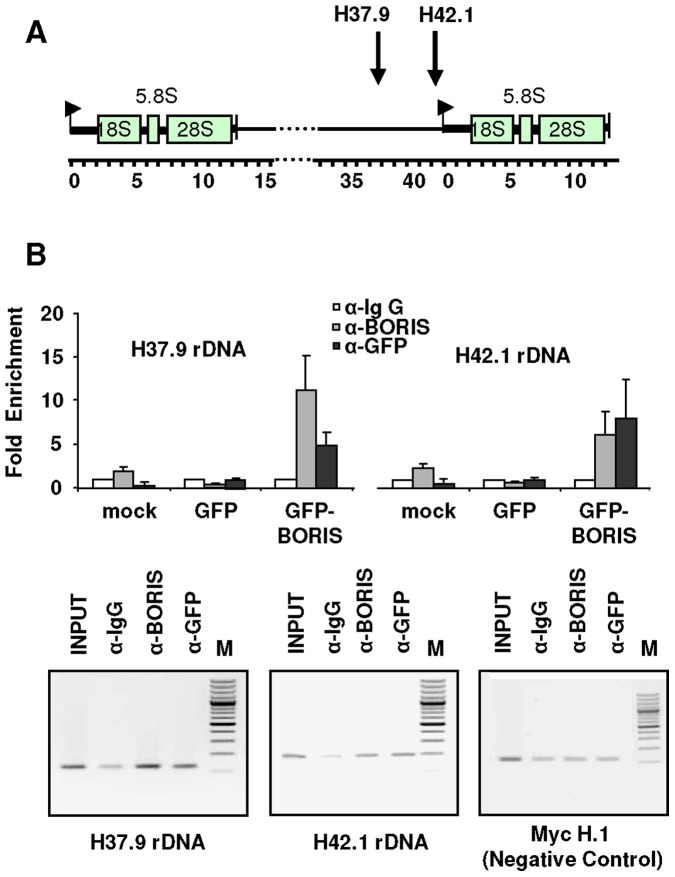
Exogenous GFP-BORIS binds ribosomal DNA. A ) Scheme showing the location of the H37.9 and H42.1 sites utilised for the studies within the ribosomal intergenic region of the rDNA repeats. **B**) In vivo binding of BORIS to ribosomal DNA (rDNA). Chromatin immunoprecipitation (ChIP) analyses with anti-BORIS (grey bars) or anti-GFP (black bars) antibodies show BORIS occupancy of H37.9 and H42.1 rDNA sites. Chromatin was prepared from HEK293T cells mock transfected or transfected with GFP or GFP-BORIS expression vectors as indicated. Relative enrichment was quantified by real-time PCR with the H37.9 and H42.1 rDNA primer sets. Data were normalised against the enrichment for the negative control Myc-H.1 [73]. The value for the amount of PCR product present from ChIP assay with anti-IgG antibody (white bars) was set as 1. Small bars are s.d. of two independent experiments performed in duplicate samples. Bottom panels show typical PCR products after the ChIP analyses.

We recently described an epigenetic regulation of ribosomal chromatin by CTCF and identified two CTCF binding sites at the intergenic region of the human rDNA repeats [39]. UBF was found to be a common interacting partner of CTCF and BORIS. Consistently, chromatin immunoprecipitation assays (CHIP) in HEK293T cells transfected with the GFP-BORIS vector showed occupancy of rDNA sites by BORIS (Fig. 4A,B). Therefore, both H37.9 and H42.1 rDNA sequences are common binding sites for CTCF and BORIS at the intergenic rDNA region.

**Figure 5:**
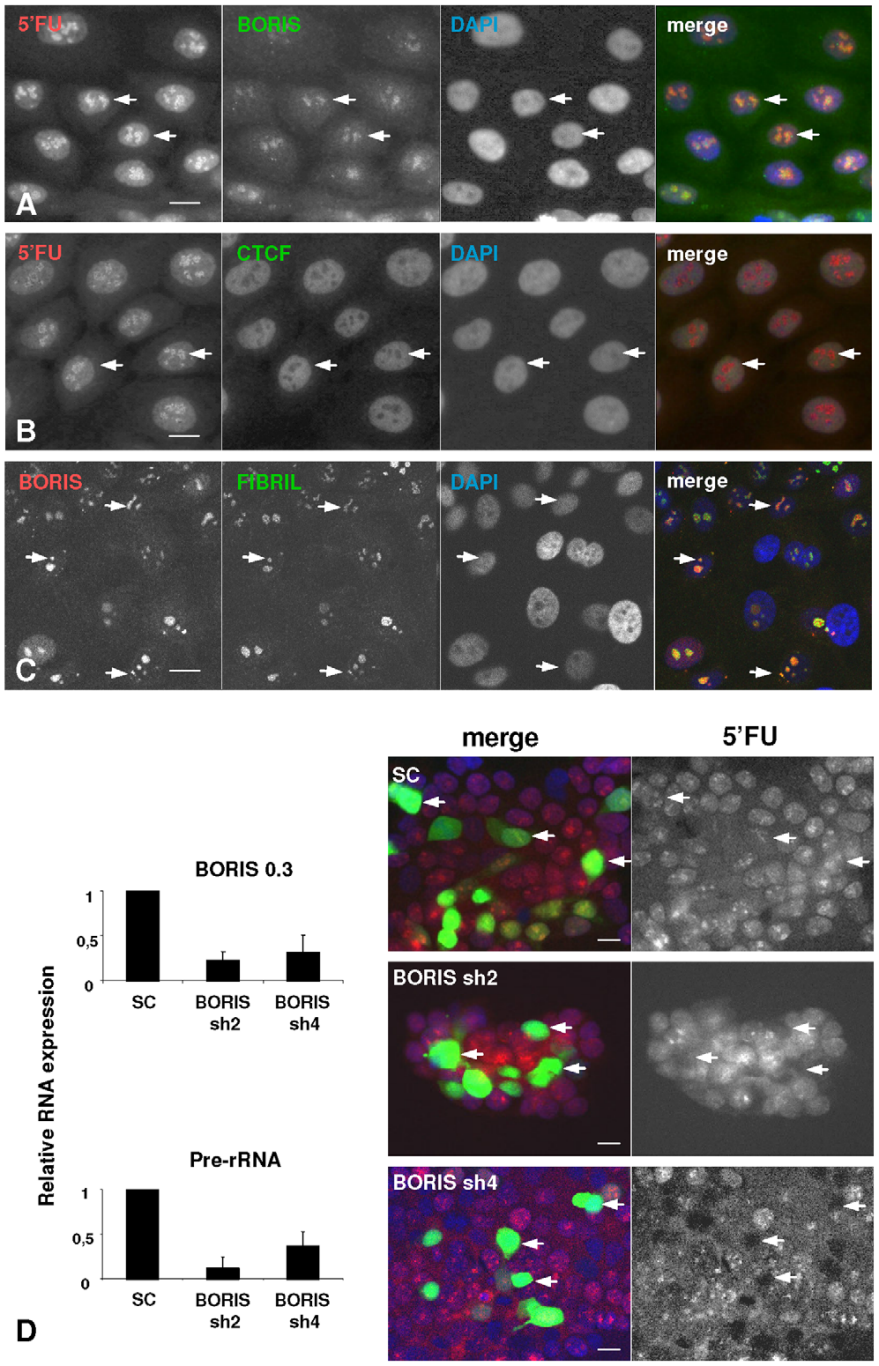
Endogenous BORIS localises to nuclear active transcription sites of primary keratinocytes. A–C ) Human primary keratinocytes were pulse-labelled with 5′fluorouridine (5′FU) for 10 min to label nascent RNA. Double immunofluorescence was performed with an anti-BrdU antibody to detect 5′FU or Fibrillarin and anti-BORIS or anti-CTCF, as indicated. Nuclei were visualised with DAPI (blue). Colours as indicated. Note that endogenous BORIS localises to nuclear areas of nascent RNA in the nucleolus and other small spots (arrows). Scale bar: 20 µm. **D**) Partial knocking-down of BORIS by transient tranfection of scrambled (SC) or specific shRNAs in HEK293T cells affects transcription. Left bar histograms: relative RNA expression by RT-PCR of BORIS or Pre-RNA after transfections; small bars are s.e.m. Right panels: pulse-labelled cells with 5′FU after transfections: GFP labels transfected cells, in green, 5′FU in red, Dapi in blue. Note that transfections with BORIS shRNAs inhibit global transcription (arrows), but not transfections with the scrambled control (arrows). Microphotographs representative of two independent experiments. Scale bar: 40 µm.

Keratinocytes can be isolated from human skin and cultured under conditions close to physiological [31]. We took advantage of these primary cultures to further investigate the localisation of CTCF and BORIS in epidermal cells. Expression of the endogenous proteins was first assessed in primary keratinocytes by western blotting and immunofluorescence as above (Information S1). The results were consistent with what observed in the epidermis. BORIS was detected in discrete areas of the keratinocyte nucleus (arrows; Information S1), whereas CTCF was evenly distributed throughout the nucleus (Information S1). To investigate the potential role of BORIS within the keratinocyte nucleus, we performed run-on transcription assays to detect nascent mRNA. This useful technique analyses overall gene transcription in live cells [37], [40], [46]. A short pulse with 5′-fluorouridine (5′FU) allows detection of foci of nascent RNA molecules. A 10 min pulse of 5′FU in keratinocytes revealed a classical pattern of transcription foci among which the nucleolus was the most prominent (due to transcription of ribosomal DNA) but not the only one (arrows; Fig. 5A). Interestingly, double staining for 5′FU and endogenous BORIS revealed a tight association not only within the nucleoli, but at all areas of nascent RNA (Fig. 5A). On the contrary, endogenous CTCF in keratinocytes did not localise to areas of 5′FU incorporation (Fig. 5B). Finally, we questioned whether BORIS foci were related to DNA replication. Labelling of DNA synthesis by a 15 min pulse of BrdU incorporation showed that the distribution of endogenous BORIS or CTCF in the keratinocyte nucleus was unrelated to DNA replication (Information S1). To test whether there was a functional role of BORIS at sites of transcription, we made use of specific shRNA to knock-down the endogenous protein. Two different shRNAs against BORIS diminished its expression with a different efficiency (Fig. 5D). They also caused an inhibition of pre-rRNA synthesis as assessed by RT-PCR and in situ overall transcription, as compared with a scrambled irrelevant RNA sequence (Fig. 5D).

### Cell Cycle-dependent Centrosomal Localisation of BORIS

**Figure 6:**
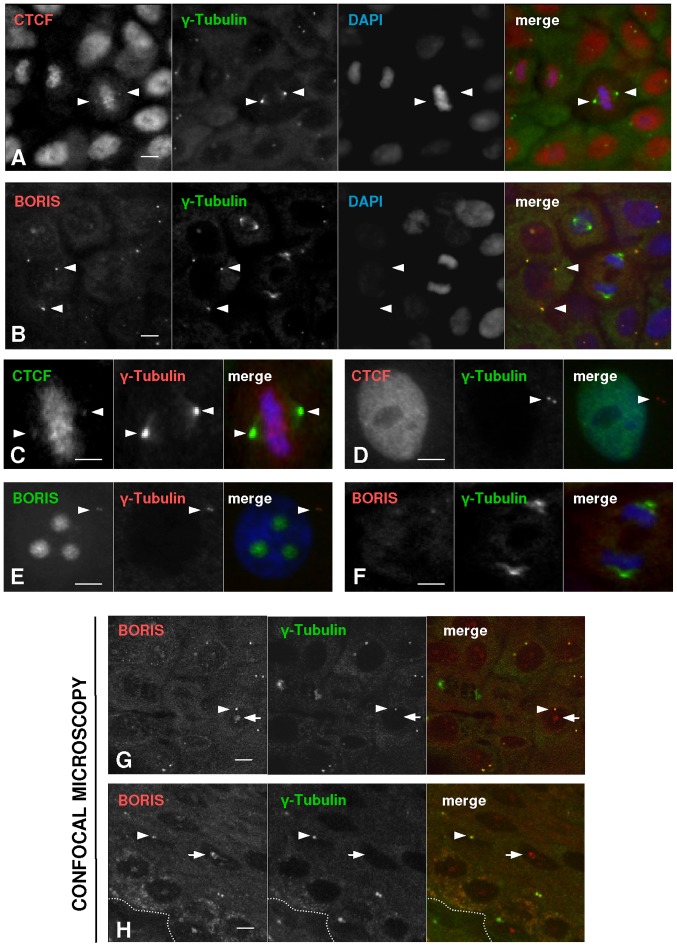
BORIS is expressed in interphase centrosomes of primary keratinocytes. A, B ) Double immunofluorescence for CTCF or BORIS (red) and centrosome marker γ-tubulin (green) on primary keratinocytes, as indicated. The nuclei were visualised with DAPI (blue) Arrowheads point at CTCF in mitotic centrosomes. Scale bar: 15 µm. **C–F**) High magnifications of keratinocyte nuclei showing either co-localisation (C,E) or not co-localisation (D,F) of CTCF or BORIS with γ-tubulin as indicated, in interphase (E,D) or metaphase cells (C,F). C and F are details of A and B, respectively. Scale bar: 10 µm. **G, H**) Double immunofluorescence and confocal microscopy analyses of primary keratinocytes (G), or skin sections (H) showing focal centrosomal co-localisation of BORIS (red) and γ-tubulin (green). Arrowheads point at BORIS in interphase centrosomes; arrows point at BORIS in the nucleolus of interphase cells. Scale bar: 15 µm.

The localisation of BORIS in a dotty perinuclear structure in the skin was reminiscent of centrosomes (see e.g., [28]). Interestingly, CTCF has been reported to localise to the centrosomes at metaphase in HeLa cells [42]. To investigate this issue, we performed double staining for CTCF or BORIS and γ-tubulin in primary keratinocytes. Centrosomes duplicate during S phase of the cell cycle and are key motors for the ordering and polar separation of the chromosomal spindle during mitosis [47]. γ-tubulin is a specific component of centrosomes [48]. We found CTCF and γ-tubulin co-localisation only sporadically in metaphase keratinocytes (Fig. 6A,C), never in interphase centrosomes (Fig. 6D). In striking contrast, BORIS was present in the centrosomes of all interphase keratinocytes (Fig. 6B,E), but not in mitotic cells as the centrosomes split far apart at metaphase (Fig. 6B,F). Detailed one-plane confocal microscopy analyses confirmed these results in primary keratinocytes and human epidermis (Fig. 6G,H). Centrosomal BORIS was undetectable at the beginning of prophase and was detected again just after cytokinesis. Similarly, BORIS centrosomal staining by confocal analyses of epidermis was strong in interphase cells (Fig. 6H) but was not detected in mitotic cells (not shown). Altogether, the results suggest a dynamic localisation of CTCF and BORIS at the centrosomes, where they alternate as the cell cycle progresses through mitosis.

**Figure 7:**
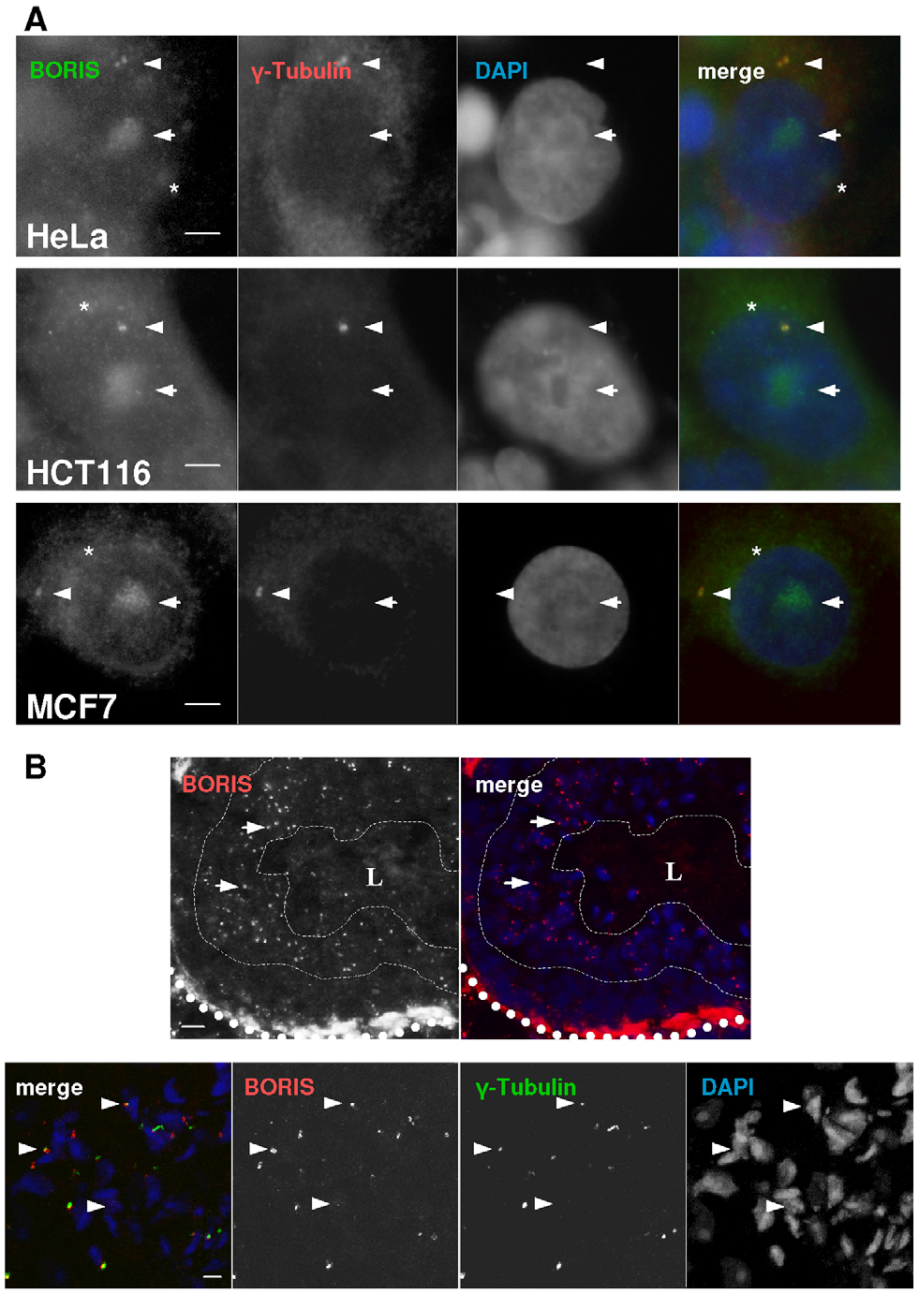
Endogenous BORIS localises to the centrosomes in human cell lines and mouse testis. **A**) Double immunofluorescence analyses were performed on the indicated human cell lines with antibodies to BORIS (green) or to γ-Tubulin (red). Nuclei were visualised with DAPI (blue). Note the distribution of BORIS in the centrosomes (arrowheads), cytoplasm (asterisks), and nucleoli (arrows). Scale bar: 40 µm. **B**) Single immunofluorescence (upper panels) or double immunofluorescence (lower panels, high magnifications) as above, on sections of mouse testis. Note a band of BORIS expression in the cyst, as described previously (upper panels, thin broken lines; Loukinov et al, 2002); thick dotted line: basal membrane of the testis cyst. Note the co-localisation of BORIS and γ-tubulin (lower panels, arrow-heads). Representative of three different biopsies from two different specimens. L: Lumen of the cyst. Scale bar: 10 µm.

Having detected BORIS in the nucleoli and centrosomes, we analysed its subcellular localisation in cell systems previously reported to express the protein (Fig. 7). We found both nucleolar and interphase-centrosomal distribution of BORIS in HeLa (cervical cancer), HCT116 (colorectal cancer) and MCF7 (breast cancer) cells. BORIS was also observed in the cytoplasm of these cancer-derived cell lines, as previously reported [17], [25], [49]. In contrast, CTCF was generally excluded from the nucleolus and interphase centrosomes in these cells (Information S1). In addition, we investigated the subcellular localisation of BORIS in mouse normal testis, where BORIS was initially detected. We observed a punctate distribution of BORIS in the testis epithelium as reported (Fig. 7B; [11]). As in human epidermis, BORIS co-localised with the nucleolar marker UBF within the nuclei (not shown) and with the centrosomal marker γ-tubulin beside the nuclei (Fig. 7B).

### BORIS Induces Accumulation of Cells in S Phase and Genomic Instability

**Figure 8:**
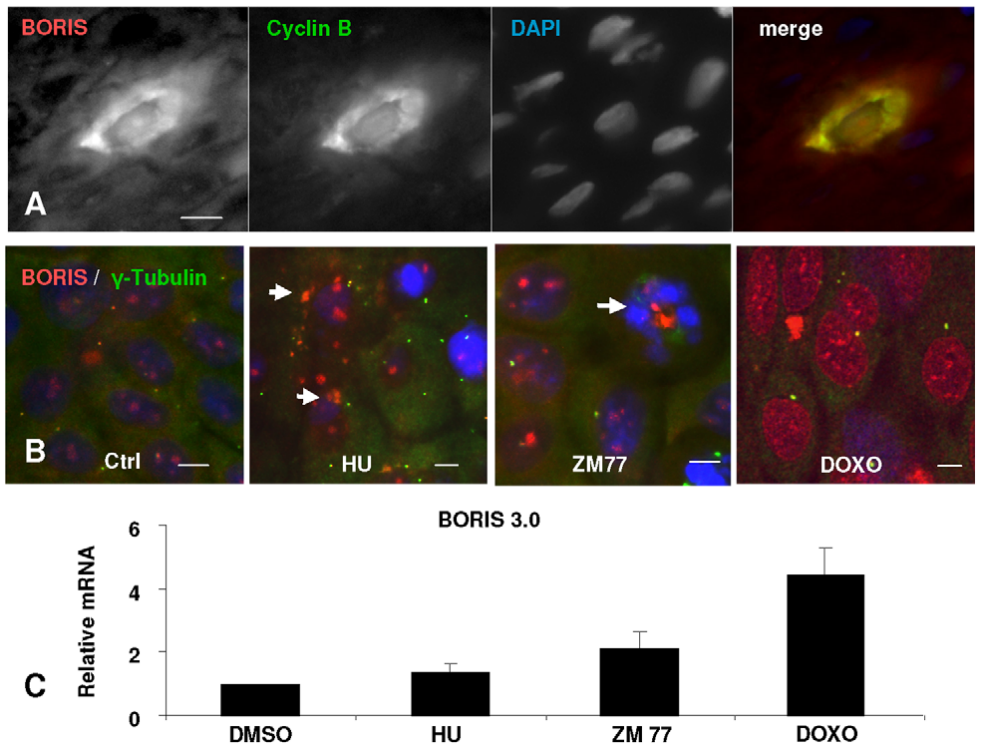
Accumulation of BORIS upon S phase and mitosis arrest. **A**) BORIS accumulates in differentiating keratinocytes of epidermis undergoing mitosis arrest as revealed by double labelling of human epidermis for BORIS (red) and Cyclin B (green); nuclear DNA (DAPI, blue). Scale bar: 15 µm. **B**) BORIS accumulates in primary keratinocytes treated for 24 h with inhibitors of the G1/S transition of the cell cycle (hydroxyurea, HU), the mitosis checkpoint (ZM77 for Aurora B kinase), or the genotoxic agent doxorubicin (DOXO). Microphotographs show merge of anti-BORIS, anti-γ-tubulin for centrosomes (green) and DAPI for nuclear DNA (blue). Scale bar: 10 µm. Arrows point at accumulation of BORIS. Note that DOXO provokes a generalised increased of BORIS. **C**) Relative BORIS mRNA as measured by RT-PCR, in primary keratinocytes treated as above. Representative of three independent experiments with cells from two different human individuals.

Epidermal keratinocytes block mitosis and accumulate mitotic cyclins as they initiate terminal differentiation [28], [32]. We have observed accumulation of BORIS in the cytoplasm of keratinocytes in differentiating layers of the epidermis that accumulate also the mitotic regulator Cyclin B (Fig. 8A). To investigate whether the accumulation of BORIS was related to a mitosis defect we blocked freshly isolated epidermal keratinocytes at mitosis by different inhibitors: i) Nocodazole, inhibitor of microtubule formation (not shown), ii) BI2536 and ZM447439 (ZM77), inhibitor of Polo-Like kinase and Aurora B kinase, respectively, that are components of the mitosis spindle checkpoint [50]. We also made use of hydroxyurea, that blocks cells at the beginning of S phase. Either blocking Mitosis or S phase progression provoked a substantial accumulation of BORIS (Fig. 8B,C, Information S1 and not shown), suggesting that the regulation of BORIS is linked to cell cycle progression. Defects in cell cycle progression accumulated BORIS and this was especially remarkable when we treated primary keratinocytes with the genotoxic agent doxorubicin (Fig. 8B,C), that induces in these cells acute DNA damage and p53 [32].

**Figure 9:**
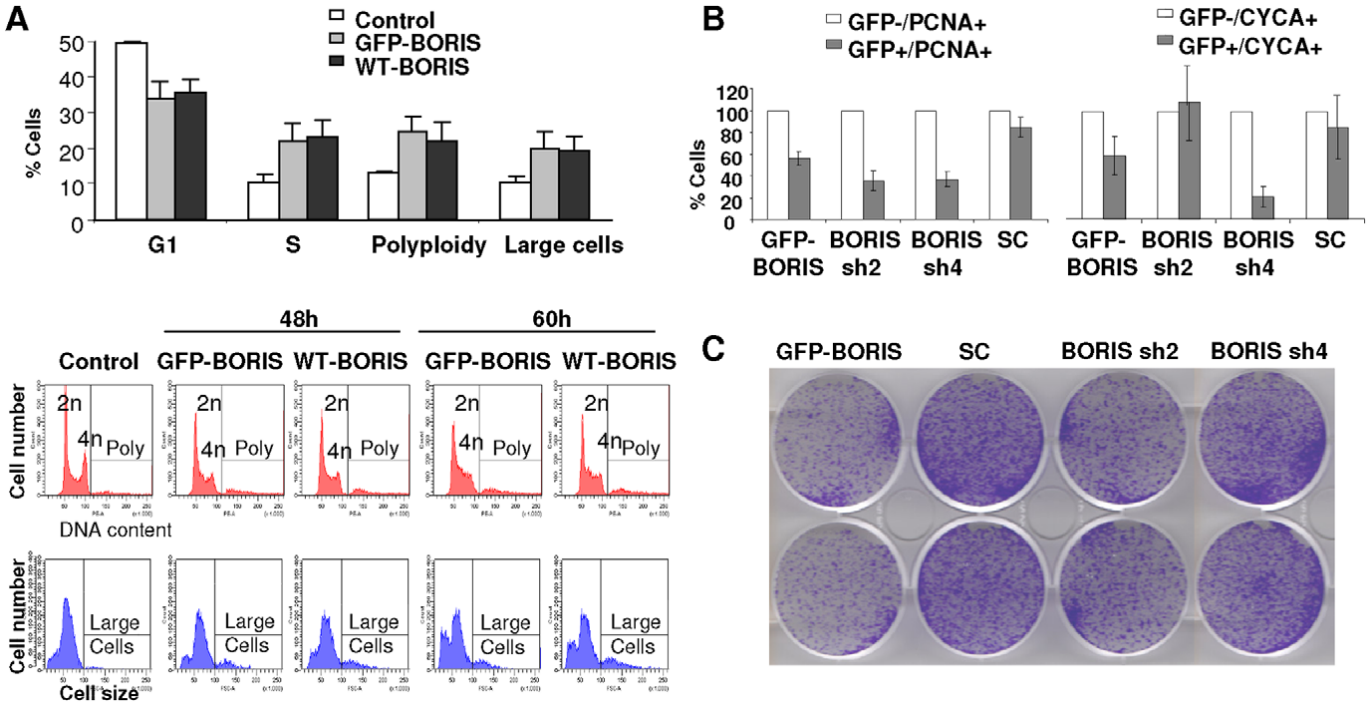
Involvement of BORIS in cell cycle progression and genomic instability. **A**) Ectopic expression of BORIS causes cell accumulation in S phase, polyploidy and cell size increase. Human embryo epithelial HEK293T cells were transfected with GFP-BORIS, the wild type gene (WT-BORIS) or no DNA (control). Bar histogram shows the quantitations of flow-cytometry analyses of two independent experiments 60 h after transient transfections. Small bars are s.d.m. The gates used for the quantitations are shown below in representative flow-cytometry histograms for the cell cycle (DNA content, in red) or cell size (Forward Scatter, in blue), 48 h or 60 h after transient transfections with the indicated genes. **B**) Transient transfection (48 h) with GFP-BORIS or scrambled control shRNA (GFP-SC) or BORIS-specific shRNAs (GFP- sh2, sh4) reduces the index of HEK293T cells expressing cell cycle markers PCNA or Cyclin A; small bars are the s.e.m. of duplicate samples. **C**) Clonogenic growth potential of HEK293T cells 7 days after transfections as above; note the decreased colony forming efficiency after transfection with GFP-BORIS or BORIS sh2.

In order to further investigate whether BORIS is involved in the progression of S phase and mitosis, we overexpressed wild type or GFP-BORIS by transient transfection in human epithelial HEK293T cells as in Fig. 3 and studied the effects on the cell cycle. As shown in Fig. 9A, overexpression of BORIS provoked an accumulation of cells in S phase and a significant increase of large and polyploid cells. Increased cell size is typical of mitosis failure and re-replication [28]. These results suggest that BORIS must be degraded in order for mitosis to progress, as other classical regulators of S phase and mitosis (e.g., Cyclins E, A and B; reviewed in [51]). To further explore whether the accumulation of cells in S phase was due to an increase of proliferation or to a cell cycle block, we performed analyses of cell cycle markers and clonal growth. Overexpression or inactivation of BORIS caused a decrease in the index of cell cycle markers PCNA and Cyclin A (Fig. 9B and Information S1) and in the clonogenic cell potential to grow (Fig. 9C). This further suggests that the regulation of BORIS is important for the correct progression of the cell cycle, its deregulation causing cell cycle defects.

## Discussion

### CTCF and BORIS in Human Epidermis

We have presented a strong body of evidence for the expression of CTCF and BORIS proteins in human epidermis. CTCF was highly ubiquitous in the epidermis although it diminished in the more differentiated layers. CTCF levels also decrease during differentiation of several hematopoietic lineages [52]. CTCF has been shown both to associate with cell growth arrest [10], [21], [22] or to stimulate proliferation (in T cells; [53]). The lower level of CTCF protein in differentiating layers of epidermis is consistent with its function in limiting cell growth, since keratinocyte differentiation involves cell size and cell mass increase [27], [28], [54].

The expression of the BORIS protein in human epidermis is unexpected since it was initially thought to be restricted to testis. However, Monk et al reported BORIS expression in ovary and oocytes [14]. Other cancer testis genes have been found in normal tissues or cells other than testis [55], [56] and therefore, low levels of BORIS might be expressed in small quantities in normal tissues. Very recently, different BORIS isoforms have been reported in somatic cells of foetal tissues including skin [15] and BORIS protein has been reported in the nucleolus of cultured cells from various origins [57]. Works studying BORIS expression and localisation have produced controversial findings [15], [58], [59]. This might be due to either low sensitivity of BORIS antibodies, to the restricted and punctate distribution of the protein in normal cells, or both [58]. Nevertheless, the distribution of BORIS in the epidermis that we have found was confirmed with three independent affinity-purified polyclonal antibodies of diverse origins (see Material and Methods). The specificity of the antibodies was also confirmed by immunofluorescence and western-blotting on cells transfected with wild type or GFP- BORIS.

### BORIS in the Nucleolus and other Transcription Sites

The nucleolus is the centre of ribosomal RNA production [60]. In the last years the nucleolus has become increasingly relevant in the control of cellular growth and oncogenesis [61]. CTCF is a predominantly nucleoplasmic protein with both nucleolar exclusion or nucleolar localisation [37], [43], [62]. CTCF function might depend on its specific localisation in the distinct nuclear compartments. We have previously reported nucleolar localisation of CTCF during erythroid differentiation [37] and in vivo binding of BORIS to the nucleolar-specific factor UBF and to ribosomal DNA [39]. In the present study we show a striking and neat accumulation of BORIS in the nucleoli, in epidermal keratinocytes and in a variety of human cancer cell lines where the protein had been previously reported to be expressed. Furthermore, exogenous GFP-BORIS accumulated in the nucleolus of human epithelial cells. While this work was being revised, [57] reported nucleolar localisation of BORIS in cultured cell lines and [63], reported changes in the architecture of the nucleolus in the absence of CTCF. BORIS and CTCF exhibit high homology in the central 11 Zinc-Finger domain. We have shown that this domain is responsible for nucleolar targeting of both CTCF [37] and BORIS (herein). Interestingly, the nucleolar transcription factor UBF is the only common interacting partner of CTCF and BORIS so far identified [39]. However, we did not clearly find endogenous CTCF within the nucleolus in our studies. Likely, the translocation of CTCF from the nucleoplasm to the nucleolus is a dynamic process, consequence of functional interactions with other macromolecules through the N- and C- terminus domains.

A similar transit at the nucleolus has been reported for other nuclear factors [64]. For instance the transcription factor MYC is rarely found in the nucleoli yet it plays an important role in the regulation of rDNA transcription. In addition, we have previously shown that CTCF goes to the nucleoli during erythroid differentiation, which involves cell growth arrest [37]. Within the epidermis, nucleolar CTCF might be present in individual cells at a very particular time and be hardly detectable. This would be in agreement with its reported function in cell growth arrest, since epidermal cells are continuously undergoing cellular growth [28], [32].

In our experiments, BORIS not only localised to the nucleolus within the nucleus. It was frequently detected in other nuclear spots in human keratinocytes both in the epidermis and in primary cultures. Labelling nascent RNA in live cells showed that the endogenous protein was present in sites of RNA transcription. Moreover, knocking-down BORIS with specific shRNAs caused a reduction in the synthesis of rRNA (RT-PCR) and global RNA (in vivo labelling). This suggests a role for BORIS in the licensing of RNA transcription.

### BORIS in the Centrosome

**Figure 10:**
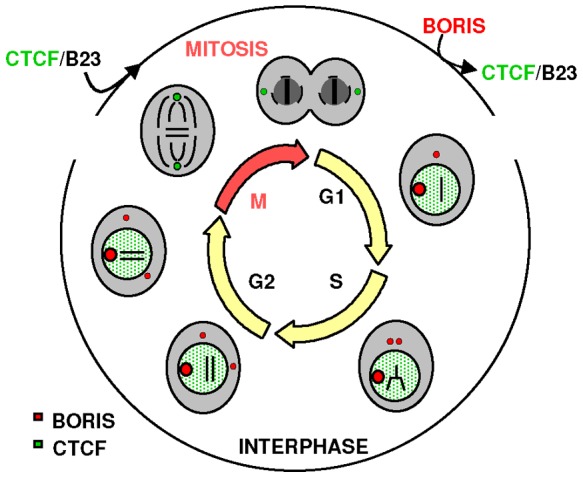
Model for the dynamics of CTCF and BORIS in the nucleus and the centrosomes during the cell cycle. CTCF is found throughout the nucleus with a granular pattern and associated with centrosomes at metaphase [42] and herein). BORIS is present in all nucleoli and in interphase centrosomes, but not in mitotic centrosomes. BORIS and CTCF appear to be mutually exclusive in the nucleus and centrosomes during the cell cycle. CTCF interacts with nucleophosmin/B23 that navigates from the nucleolus to the centrosomes, possibly to coordinate S phase (duplication and separation of centrosomes) with mitosis (centrosome polarisation and stretching; Okuda, 2002).

BORIS localised in the centrosomes in the epidermis of skin section and in primary keratinocytes, as revealed by co-localisation with the specific centrosome-specific marker γ-tubulin. We also found BORIS in the centrosomes of mouse testis and a variety of human non-tumour or cancer cell lines of different origins. BORIS was present in the centrosomes up to prometaphase, when they split far apart and then it became undetectable (Fig. 10). Centrosomes duplicate during S phase and split apart at the beginning of mitosis [65]. Through the control of microtubule nucleation, they are involved in the assembly and organization of the mitotic bipolar spindle that ensures accurate chromosome segregation. The subcellular distribution of BORIS in both nucleoli and centrosomes might seem intriguing. However, proteins with a similar dual distribution have been described previously and might have a role in coordinating S phase with mitosis [66], [67]. For instance, they can inhibit centrosome duplication until the S phase is completed. One of these proteins is nucleophosmin/B23, which is involved in ribosome biogenesis and localises to the centrosome to prevent it from duplication (Fig. 10). They might thus contribute to maintain genome integrity.

Interestingly, CTCF has been reported to localise to metaphase centrosomes [42], [43] and to interact with nucleophosmin/B23 [68]. Therefore both CTCF and B23 are detected in centrosomes from metaphase to G1 phase, precisely when BORIS is not there detectable. This alternate pattern of CTCF and BORIS association with the centrosomes may be important for their function during the cell cycle (Fig. 10).

### Potential Functions of BORIS

Despite BORIS being expressed in a variety of human malignancies, little is known about its biological functions. This issue has in part been hampered by the fact that detection of BORIS was initially restricted to testis [11]. The detection of BORIS in normal epidermis provides new insight.

BORIS and CTCF are thought to have opposed functions [10], [11]. Although they have a highly conserved DNA binding domain and thus they are thought to bind to the same sites, they are often expressed in a different manner. CTCF is ubiquitous in normal tissues, has cell growth restrictive activities and is often lost in cancer [20]. In contrast, BORIS is detected in cancer and in immortalised cell lines, suggesting that it associates with cell growth [10], [11]. This may explain why tissues with a high cell turn-over (such as testis and epidermis) express more detectable levels of BORIS. Interestingly, within the epidermal nuclei CTCF accumulated mainly around regions of heterochromatin whereas BORIS localised to areas of euchromatin, on basis of the DNA and histone density. Euchromatin is formed by decondensed chromatin required to allow DNA replication or transcription.

Considering the presence of BORIS in euchromatin domains, the evidence involving the endogenous protein in global transcription and the role of CTCF in chromatin remodelling, it is tempting to speculate that BORIS might participate in the unfolding of the chromatin preceding transcription. This would explain why BORIS is undetectable during mitosis, when chromatin is highly condensed. Within the same lines, when we partially inhibited the endogenous BORIS protein by shRNAs, RNA transcription was significantly inhibited. This model is consistent with the frequent overexpression of BORIS in cancer cells [60]. It is unclear what drives the two proteins to different sites according to the different needs. Some studies have found an association between CTCF and BORIS DNA binding and the chromatin status (reviewed in [69]). It is conceivable that DNA methylation, specific histone modifications and different binding partners may influence the binding of CTCF or BORIS to the chromatin [39] and this is an issue to pursue. Within the same lines, the state of DNA methylation and histone modifications has been found to change during epidermal differentiation [29].

Overexpression of BORIS caused an accumulation of cells in S and G2/M phases of the cell cycle. This effect was unrelated to an increase of the cell cycle and proliferation. First, there was no apparent association between endogenous BORIS and sites of DNA replication. Second, the proportion of cells expressing cell cycle markers PCNA or Cyclin A diminished after transfection with BORIS, as well as the capacity to grow and form colonies. The accumulation of cells in S phase and mitosis upon overexpression of BORIS might be consequence of replication defects induced by altered chromatin unfolding and/or defects in coordinating S phase with mitosis. During the cell cycle, mitosis must not start before the S phase is completed and S phase regulators are degraded [51]. BORIS might thus have a role in coordinating S phase with mitosis and when deregulated, in genomic instability. Consistently, in our experiments ectopic expression of BORIS caused polyploidy, a marker of mitotic failure and genomic instability. Moreover, the inhibition of endogenous BORIS by shRNAs caused a decrease in the cell cycle index and in the clonogenic capacity. Other lines of evidence further support a role of BORIS in genome instability:

- in our experiments, BORIS accumulated in the cytoplasm of keratinocytes upon a mitosis block caused by differentiation or by inhibitors of Aurora B and Polo-like kinase, members of the spindle checkpoint [65];
- the most striking accumulation of BORIS in keratinocytes occurred in response to doxorubicin, that causes DNA damage, and proteins involved in the DNA damage checkpoint have been found in the centrosome [70].
- within the epidermis, BORIS accumulated with mitotic Cyclin B in the cytoplasm of suprabasal cells arrested in mitosis;
- BORIS accumulates in the cytoplasm of pre-meiotic spermatocytes and when absent in mice it provokes defects in spermatogenesis [11], [16].
- CTCF has been shown to interact with cohesins [71], proteins that hold sister chromatids together during metaphase. Interestingly, cohesins have recently been found in the centrosomes, to keep them together until they separate before mitosis [72].
- inactivation of CTCF and the guardian of the genome p53, provokes strong activation of BORIS [25].

In summary, optimal levels of BORIS may be needed to support normal cell division. Conversely, defects in cell division may lead to the accumulation of cytoplasmic non-functional BORIS. CTCF and BORIS might thus antagonise each other as the different needs for chromatin folding or unfolding and centrosome duplication and separation succeed during the cell cycle (Fig. 10). They also might have a role in linking these events with centrosome duplication and mitosis. This model is consistent with the proposed activity of CTCF as a tumour suppressor and the frequent deregulation of BORIS in cancer.

## Supporting Information

Information S1 **Figures 1 – and Table I.**
(PDF)

Video S1. **Video fragment showing the co-localisation of GFP-BORIS and Fibrillarin-Cherry in live cells after transient transfection (freeze frames in** **Fig. 3J** **).** Representative of 20 different fields of two independent experiments. Acq. Time: hours after transfection.
(WMV)
