# Supplementary material for: A Cell Cycle Role for the Epigenetic Factor CTCF-L/BORIS
Source: PLoS One. 2012 Jun 19;7(6):e39371. doi: 10.1371/journal.pone.0039371 (PMC3378572; doi:10.1371/journal.pone.0039371)
Supplement: Information S1 — Figures 1 – and Table I. (PDF) [file pone.0039371.s001.pdf]

Supporting Information S1

A Cell Cycle Role for the Epigenetic Factor CTCF-L/BORIS.

Manuel Rosa-Garrido, Laura Ceballos, Pilar Alonso-Lecue, Cristina Abraira, M. Dolores Delgado and Alberto Gandarillas.

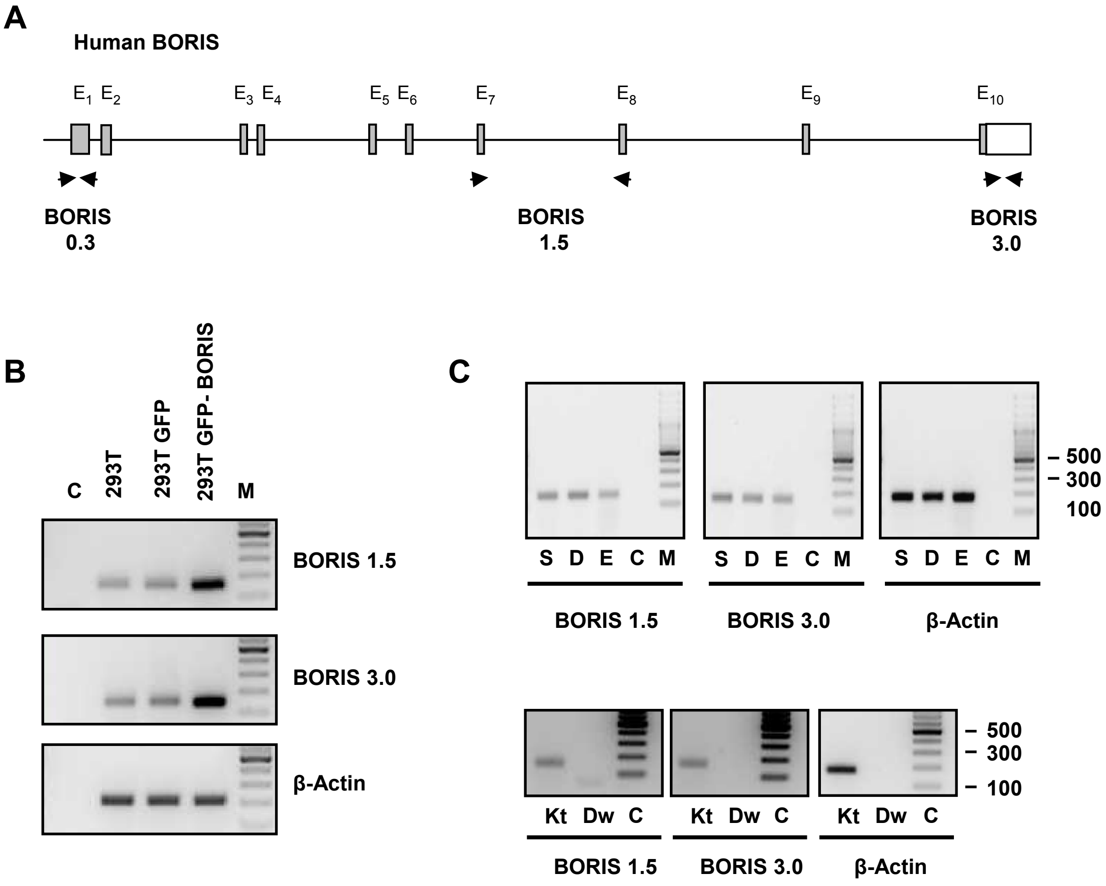

Supplementary Figure 1. **RT-PCR analyses for BORIS mRNA expression. A)** Scheme of the human BORIS gene showing the localisation of the primers used for the RT-PCR analyses. **B)** RT-PCR assays for BORIS mRNA in human skin and primary keratinocytes using primers H1.5 and H3.0 **C)** RT-PCR assays for BORIS mRNA in HEK 293T cells transfected with GFP or GFP-BORIS using primers H1.5 y H3.0.

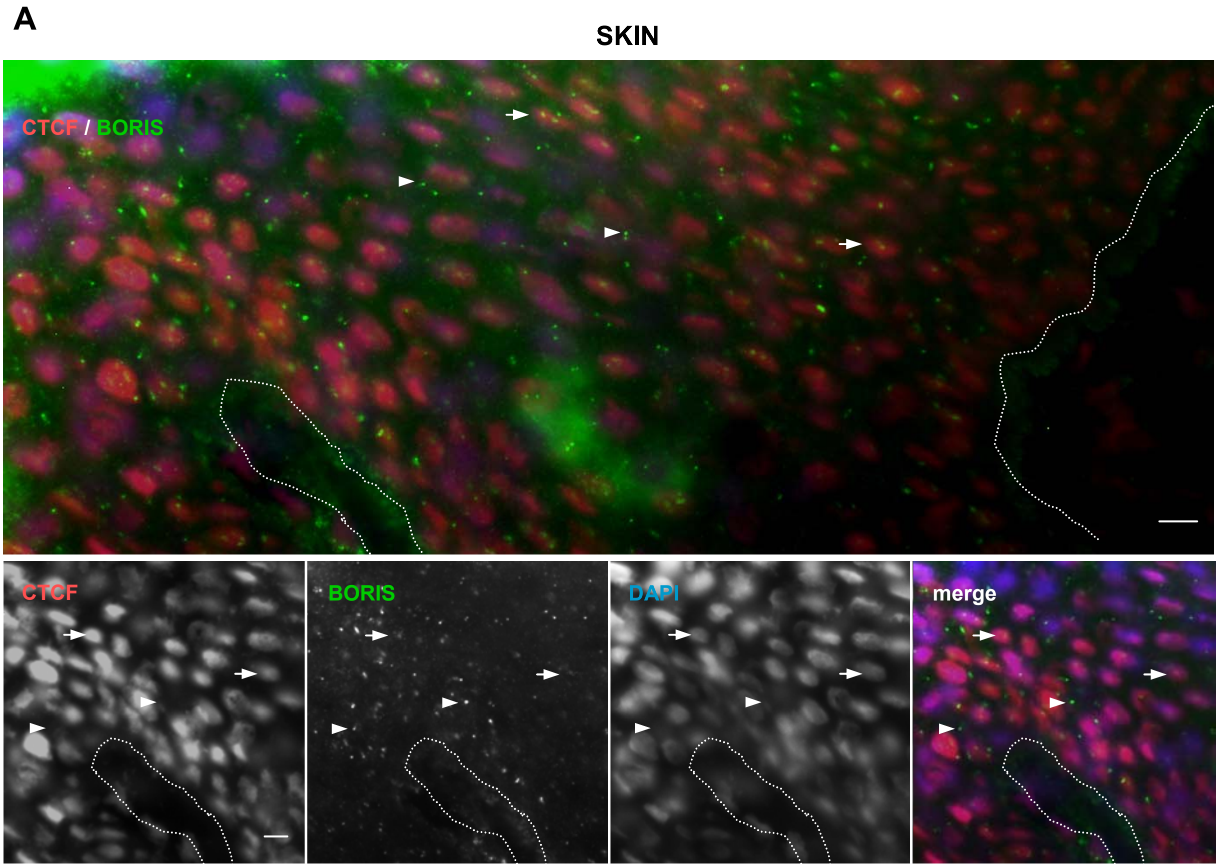

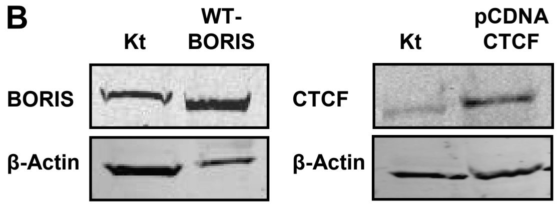

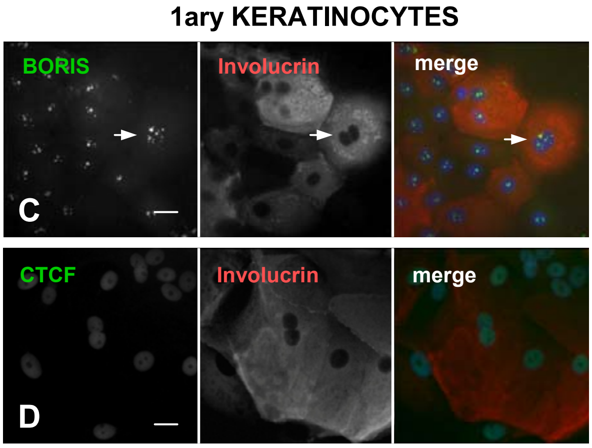

Supplementary Figure 2. Distribution of BORIS and CTCF in human epidermal cells. A) Double immunofluorescence analyses on human skin sections with antibodies to BORIS (green) and CTCF (red) and Dapi (blue). Lower panels are a detailed region of the upper panel. Note that BORIS and CTCF do not generally co-localise in the epidermis. Arrows: BORIS within the nucleus; arrowheads: BORIS dots beside the nucleus. Scale bar: 50 μm. **B)** BORIS and CTCF protein expression by western blotting in primary keratinocytes (Kt) or in HEK293T cells transfected with WT-BORIS or pCDNA-CTCF vectors as positive controls. β-Actin was a loading control. **C, D)** BORIS and CTCF in primary keratinocytes. Double immunofluorescence analyses for BORIS or CTCF (green, the cytoplasmic differentiation marker involucrin (red) and Dapi (blue), as indicated. Arrow points at BORIS spots in the nucleus of an involucrin-positive cell. Scale bar: 40 μm.

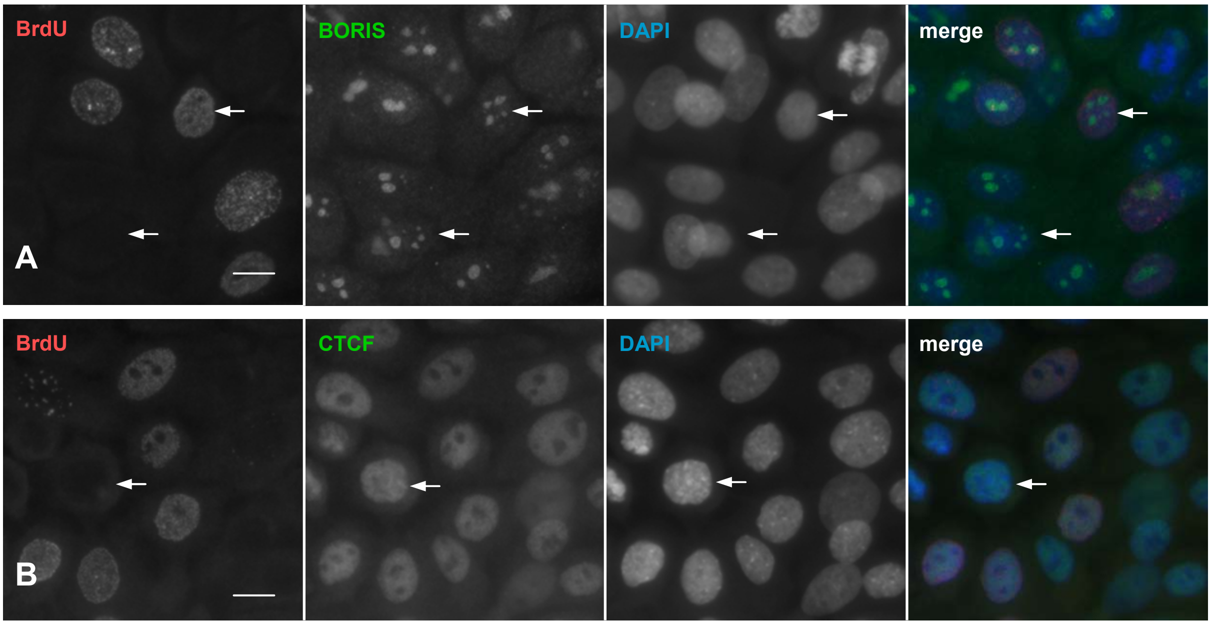

Supplementary Figure 3. A,B) Bromodeoxyuridine (BrdU) incorporation in live primary keratinocytes to detect newly synthesised DNA. Keratinocytes were pulse-labelled with BrdU for 15 min and detected by immunofluorescence (red) in double labelling with anti-BORIS (green in A) or anti-CTCF (green in B) antibodies. A report between either protein and DNA synthesis was not observed (arrows). Nuclei were visualised with DAPI (blue). Scale bar: 20 μm.

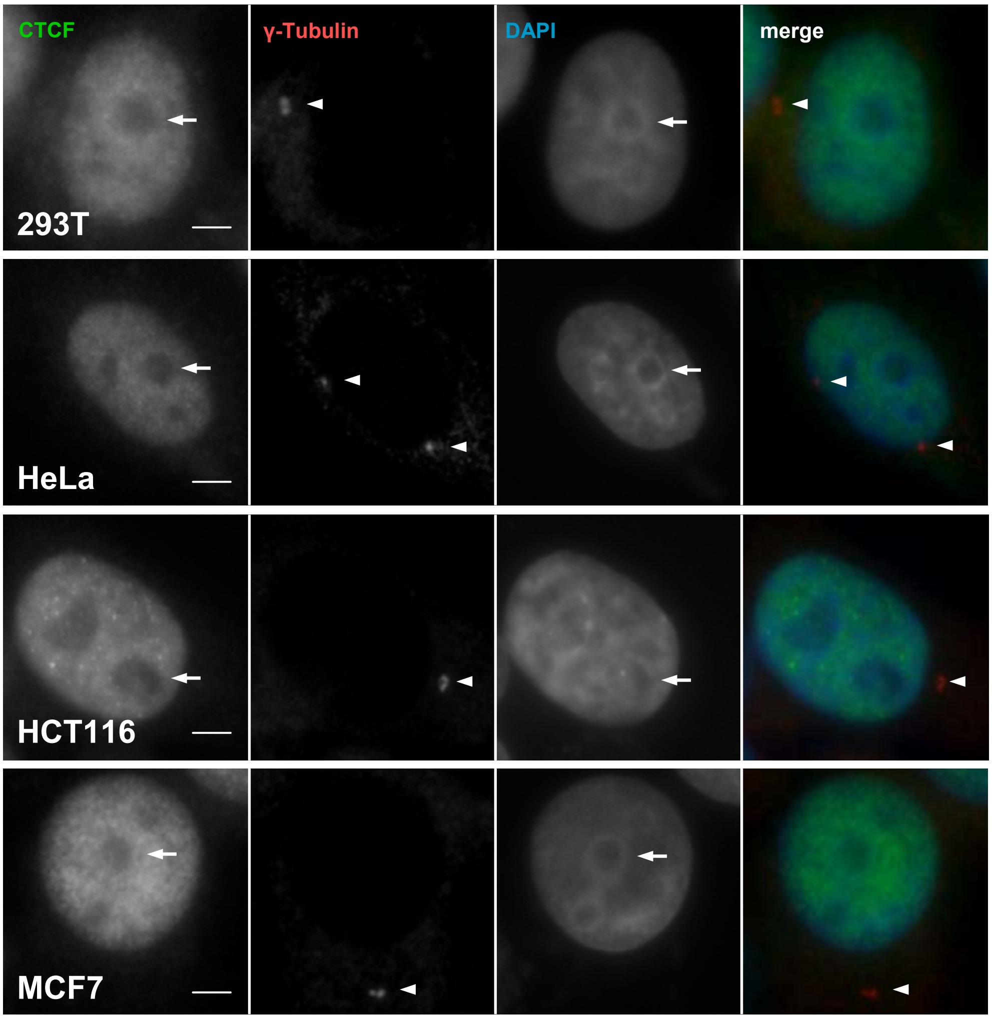

Supplementary Figure 4. Subcellular localisation of CTCF in human cell lines of various origins. Double immunofluorescence was performed on the indicated human cell lines for CTCF (green) and γ-tubulin (red), a centrosomal marker. Nuclei were visualised with DAPI (blue). Merge images show the distribution of CTCF throughout the nucleoplasm. Note that unlike BORIS (Fig. 7), CTCF is generally excluded from the nucleoli (arrows) and does not co-localise with γ-tubulin in interphase centrosomes (arrow-heads). Scale bar: 5 µm.

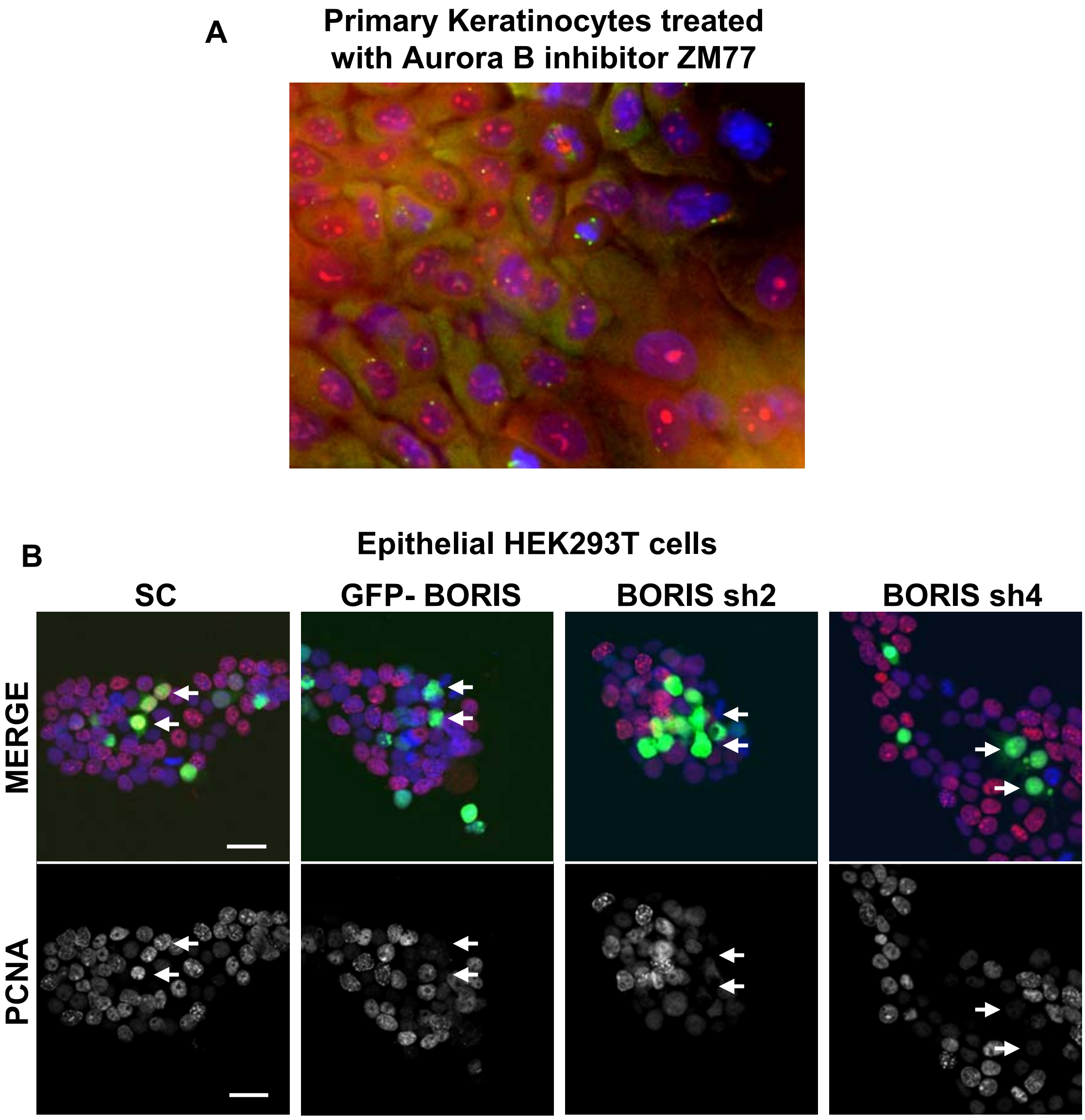

Supplementary Figure 5. BORIS and the cell cycle: additional details to Figs. 8 and 9. A) Entire field of amplified detail in Fig. 8B showing the accumulation of BORIS (arrows) caused by ZM77, inhibitor of the spindle mitosis checkpoint component Aurora B. Scale bar: 10 μm. **B)** Representative microphotographs of immunofluorescence studies quantitated in Fig. 9B, showing the reduced index of cell cycle marker PCNA in HEK293T cells transfected with GFP-BORIS or BORIS shRNAs (GFP reporter). SC: scrambled control shRNA. Arrows indicate examples of transfected cells. Scale Bar: 40 μm.

Supplementary Table I. Primers used for RT-PCR analyses

Amplified gene	Name	Primers	Amplicon size
Human BORIS	BORIS H 0.3	5´-TGCAGGATATGAGCTTGCTG-3´<br>5´-CTAATGGCCACACACTGCTG-3´	130 bp
Human BORIS	BORIS H 1.5	5´-CTGCAAGCAGGAACGTCATA-3´<br>5´-TGCATCGTGGTATTTCCTGA-3´	130 bp
Human BORIS	BORIS H 3.0	5´-TTTCCCTTTTGCCAGTTGAC-3´<br>5´-AGGTTGGCAGCAAAACAAAC-3´	145 bp
Human CTCF	CTCF	5´-TTACACGTGTCCACGGCGTTC-3´<br>5´-GCTTGTATGTGTCCCTGCTGGCA-3´	268 bp
Human β-Actin	ACTIN	5´-AAAATCTGGCACCACACCTTC-3´<br>5´-TAGCACAGCCTGGATAGCAA-3´	171pb
Mouse BORIS	BORIS Ms 0.3	5´-ACGACGAAATTGTCCTGACC-3´<br>5´-GAGCTTGGAGGAAGTGAACG-3´	179 bp
Mouse BORIS	BORIS Ms 0.8	5´-GACGGGGTACAAAGAGTGGA-3´<br>5´-TCTGGTACCGTCCCTGAAAG-3´	215 bp
Mouse GAPDH	GAPDH	5´-CGACATACTCAGCACCGGC-3´<br>5´-GGGAAGCCCATCACCATCT-3´	110 bp
